# Modeling time-varying efficacy of RTS,S/AS01 malaria vaccine across trial settings

**DOI:** 10.1038/s41541-026-01519-4

**Published:** 2026-07-03

**Authors:** Ziwei Zhao, Paul Milligan, Yin Bun Cheung

**Affiliations:** 1 Centre for Biomedical Data Science, Duke-NUS Medical School, National University of Singapore, Singapore, Singapore; 2 Faculty of Epidemiology and Population Health, London School of Hygiene & Tropical Medicine, London, UK; 3 Signature Programme in Health Services & Systems Research, Duke-NUS Medical School, Singapore, Singapore; 4 Tampere Center for Child, Adolescent and Maternal Health Research, Tampere University, Tampere, Finland

**Keywords:** Computational biology and bioinformatics, Diseases, Health care, Immunology, Mathematics and computing, Medical research

## Abstract

Correctly evaluating how vaccine efficacy (VE) changes over time is important for understanding long-term protection and guiding vaccination strategies. Using the 18-month pre-booster data from the multi-center RTS,S/AS01 malaria vaccine trial, we examined how different approaches of modeling time-varying VE affect the interpretation of vaccine protection. Four commonly used linear-in-parameter functions and a four-parameter monotonic function were evaluated and compared with semi-parametric spline benchmarks. Across study sites, the four-parameter function was favored when a sufficient number of clinical malaria episodes were observed. Different VE specifications led to substantially different estimates of vaccine impact under a seasonal delivery strategy. Across settings with a sufficient number of episodes, the four-parameter model recovered an inverse-sigmoid pattern of RTS,S/AS01 protection over 18 months, with rapid early decline followed by stabilization at 20-40%. These findings show that modeling choices influence interpretation of vaccine protection and support the four-parameter approach as a parsimonious, biologically-interpretable parametric form for characterizing waning VE in the RTS,S/AS01 setting, with broader applicability across other vaccine platforms requiring further evaluation.

## Introduction

Protective efficacy of vaccines tends to wane over time, as has been observed for malaria, rotavirus, and COVID-19 vaccines^1–3^. Understanding how vaccine efficacy (VE) changes following immunization is therefore essential for assessing the durability of protection, comparing vaccine performance across settings, and optimizing vaccination schedules. In clinical studies of infectious disease prevention, conventional approaches often summarize VE as one minus a constant hazard ratio (HR) estimated from Cox-type proportional hazard (PH) models^4–6^. When efficacy wanes, the PH assumption is violated, and the resulting constant VE estimates can become sensitive to trial implementation features such as study duration, sample attrition, or delivery strategies in regions with seasonal disease transmission^7^. These limitations have motivated increasing interest in explicitly modeling the time-varying VE curve to better reflect underlying protection dynamics.

Several approaches have been proposed to capture the time-varying VE curve, including using step functions, splines, and parametric functions^8–15^. Recently, the area under the VE curve (AUC) has been proposed as a robust summary measure of time-varying efficacy^7,10^. By integrating VE over time, the AUC summarizes vaccine protection over a specified follow-up period into a single value and has been shown to be less sensitive to trial implementation features than constant VE estimates from PH models. This AUC-based method can be used to estimate vaccine impact in terms of number of cases averted (NCA) under different vaccine delivery strategies, such as seasonal or age-based. Seasonal vaccination has been proposed for respiratory syncytial virus^16^ and is routine for influenza globally^17^.

Summarizing VE into a single measure does not eliminate the need to accurately specify the underlying shape of the VE curve. A well-specified VE curve can provide important information about the initial level of protection, the rate of decline, the presence of a long-term plateau, and the implications for booster timing^1,18,19^. Different assumptions about VE shape can therefore lead to different interpretations of durability and different NCA estimates for vaccine impact, even when overall summary measures are similar.

Previous vaccine studies relied on simple linear-in-parameter functions to specify the shape of the VE trajectory, such as *t*, $\sqrt{\mathrm{t}}$, log(*t*), and 1/*t*^1^. These functional forms produce monotonic VE(*t*) trajectories of distinct shapes: $\sqrt{\mathrm{t}}$ gives the most gradual, approximately linear decline; *t* declines slowly at first and then more steeply over time; log(*t*) and 1/*t* show most of their decline in the early months before flattening, most sharply for 1/*t* (Fig. S1). The particular shape of the trajectory depends on fitted parameters, so a given functional form can produce other patterns. For example, with a sufficiently large coefficient, the 1/*t* form gives a stable initial plateau followed by a steady decline rather than a rapid early drop. These simple linear-in-parameter functions are straightforward to fit but still very limited in flexibility. Splines are highly flexible but often lack interpretability and generalizability across settings^12–14^. A flexible and interpretable four-parameter monotonic function has been introduced in malaria chemoprevention research. It shows the potential to capture both the rapid initial decline and the slower long-term plateau (Fig. S1), but it has not yet been evaluated in a vaccine context, and its performance across diverse trial settings remains unclear^15^. In practice, the choice of parametric form for modeling the time-varying VE curve is typically ad-hoc, guided by model fit within individual datasets. It remains unclear whether there exists a parametric form of the VE curve that is widely usable across diverse trial settings.

In this study, we evaluated how different parametric functions for modeling time-varying VE curves influence understanding of vaccine protection and estimation of vaccine impact. We use data from the multi-center phase 3 trial of the RTS,S/AS01 vaccine. The trial evaluated the efficacy of 3- and 4-dose schedules against clinical malaria caused by *Plasmodium falciparum* in young children. Our analysis focuses on the waning efficacy during the 18-month follow-up following administration of the third dose and prior to administration of the fourth dose. We compare commonly used linear-in-parameter functions with the four-parameter monotonic function, assess their model performance across sites with varying transmission intensity, and evaluate implications for estimating NCA under a seasonal vaccination strategy.

The RTS,S/AS01 trial was conducted in sub-Saharan Africa, where malaria is holoendemic and remains a leading cause of childhood morbidity and mortality. Infants and children aged under 5 years carry a disproportionate share of this burden. Malaria seasonality in Africa is primarily driven by rainfall, with the main burden occurring during and shortly after seasonal rains in West and Central Africa, and in parts of East and Southern Africa, while transmission is year-round in equatorial areas. *P. falciparum* is responsible for over 90% of all malaria cases in this region, and most people are almost continuously infected by *P. falciparum*^20^. Earlier analyses of the RTS,S/AS01 trial described the waning pattern of VE as an initial high level of protection followed by a decline toward a lower sustained level^19,21,22^. In this work, by linking statistical performance with epidemiological interpretations, we assessed whether a single parsimonious and interpretable parametric form can recover it across diverse trial settings in sub-Saharan Africa, in place of the ad-hoc functional choices used previously, and to examine the consequences of this modeling choice for estimating vaccine impact.

## Results

### RTS,S/AS01 data

**Fig. 1:**
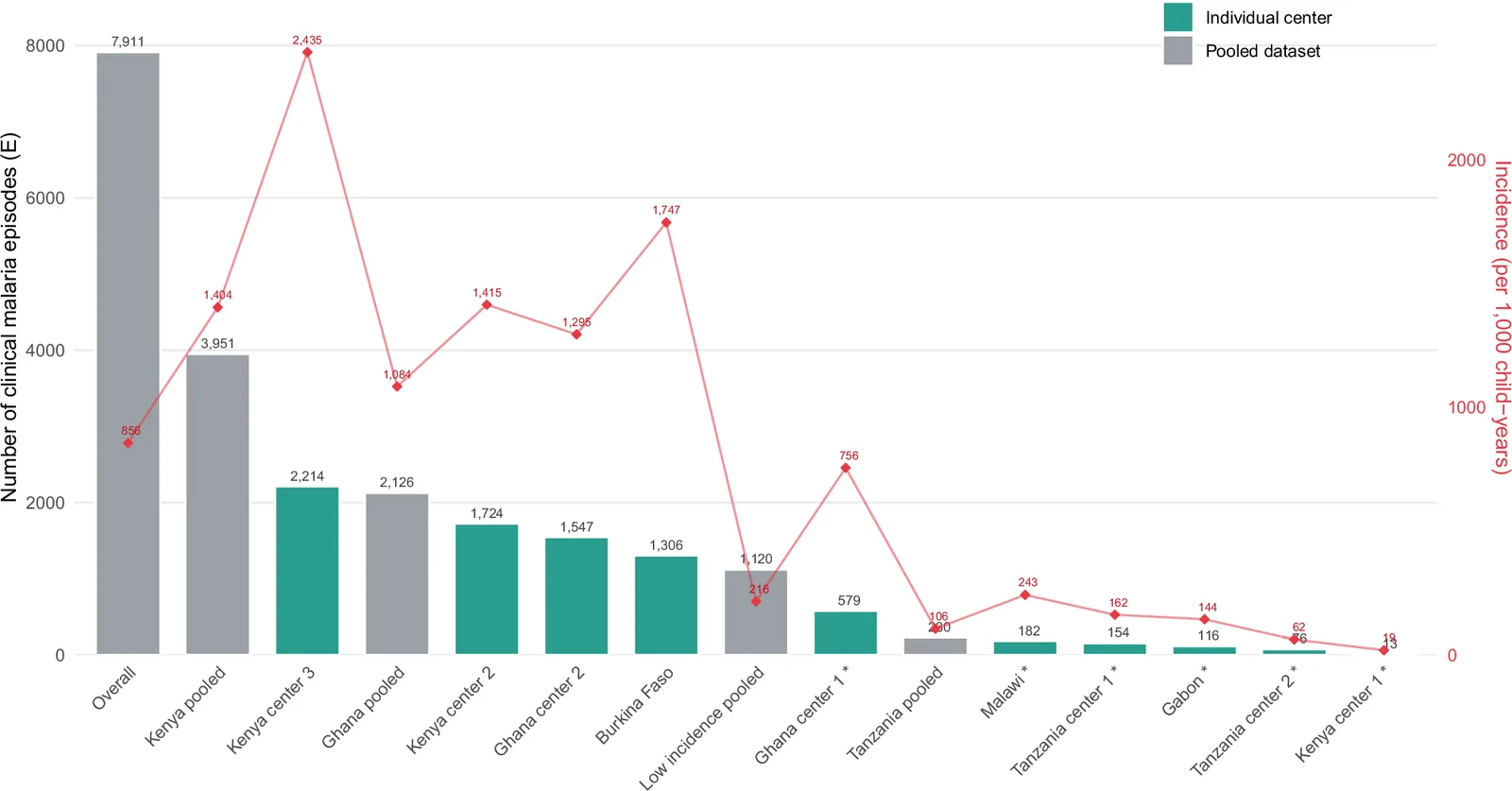
Number of clinical malaria episodes and incidence across 15 analytical datasets. Bars show the number of clinical malaria episodes (*E*, left axis) for each analytical dataset, ordered left to right by descending episode count. Green bars denote individual study centers and gray bars denote pooled datasets (the overall dataset, the three country-level pooled datasets, and the low-incidence pooled dataset). Red diamonds show the corresponding malaria incidence (right axis; clinical malaria episodes per 1000 child-year). Asterisks (*) mark the six lower-incidence centers combined in the low-incidence pooled dataset (Kenya center 1, Ghana center 1, Tanzania center 1, Tanzania center 2, Malawi, and Gabon). The ordering by episode count does not coincide with the ordering by incidence: datasets with the most episodes are not necessarily those with the highest transmission intensity.

We re-analyzed data from the phase 3 randomized controlled trial of the RTS,S/AS01 malaria vaccine for the children cohort. All analyses were restricted to the 18-month period following completion of the primary three-dose series and prior to booster vaccination. Data from 10 study centers were available in the per-protocol efficacy analysis for children. We estimated the time-varying VE separately for each study center, for pooled analyses of each country that had multiple participating centers (Tanzania, Ghana, and Kenya), and overall. To explore whether findings may differ due to a small number of malaria episodes in datasets versus due to low malaria incidence at the study sites, we also conducted a pooled analysis of 6 study centers with low malaria incidence (Ghana center 1, Malawi center, Tanzania center 2, Gabon center, Tanzania center 1, Kenya center 1), such that the total number of malaria episodes in the pooled analysis of low-incidence centers was over one thousand. All analyses that pooled multiple study centers were adjusted for center effects by Cox model stratification, which has a separate baseline hazard per stratum. Table 1 and Fig. 1 summarize and illustrate the analytical datasets information. We denote N as the number of participants and E as the number of clinical malaria episodes. The mean cumulative number of clinical malaria episodes per child by trial arm, estimated with the Nelson-Aalen estimator for recurrent events, is shown in Supplementary Figs. S2 (datasets with *E* > 1000) and S3 (*E* ≤ 1000).

**Table 1:** RTS,S/AS01 re-analysis analytical datasets summary

Analytical dataset	*N*	*E*	Incidence	Follow-up > 16 months (%)
Overall	6851	7911	855.7	94.5
Kenya pooled	2154	3951	1404.1	92.2
Kenya center 1*	510	13	19.0	94.1
Kenya center 2	919	1724	1414.7	91.8
Kenya center 3	725	2214	2434.6	91.2
Ghana pooled	1466	2126	1084.3	96.0
Ghana center 1*	558	579	755.9	97.7
Ghana center 2	908	1547	1294.8	95.0
Tanzania pooled	1533	230	106.0	97.1
Tanzania center 1*	685	154	162.4	94.9
Tanzania center 2*	848	76	62.3	98.9
Burkina Faso	580	1306	1746.8	93.8
Malawi*	541	182	242.6	93.7
Gabon*	577	116	144.3	94.1
Low incidence pooled	3719	1120	216.4	95.8

Datasets are listed with the overall dataset first, then each multi-center country (the country’s pooled row followed by its constituent centers), then the single-center countries, and finally the low-incidence pooled dataset. *N*: number of children in the per-protocol population, combining the RTS,S/AS01, and control arms. *E*: number of clinical malaria episodes after dose 3 and before booster dose, counting all recurrent episodes contributed by each child. Incidence: clinical malaria episodes per 1000 child-year in the per-protocol population. Follow-up >16 months (%): percentage of children with at least 16 months of follow-up after dose 3. Asterisks (*) mark the six lower-incidence centers combined in the low-incidence pooled dataset (Kenya center 1, Ghana center 1, Tanzania center 1, Tanzania center 2, Malawi, and Gabon).

### Model comparison

We modeled 18-month RTS,S/AS01 efficacy since dose 3 against all clinical malaria episodes, defined as an illness with axillary temperature ≥37.5 °C and *P. falciparum* asexual parasitaemia >5000 parasites/μL, or a case meeting the severe-malaria definition. We used the Andersen–Gill extension of the Cox model^5^ for recurrent events (details in “Methods”), so that each child could contribute to more than one episode. Several commonly used parametric specifications for VE(*t*) were considered, including the conventional PH model (which assumes a constant VE over follow-up), simple linear-in-parameter functions (*t*, $\sqrt{\mathrm{t}}$, log(*t*), and 1/*t*), and a flexible four-parameter monotonic function (4-par) that allows for an initial decline followed by a plateau. Estimated VE(*t*) trajectories from parametric models were further compared with spline-based estimates as a semi-parametric reference.

Model fit for each candidate function was evaluated and compared using the Akaike information criterion (AIC) across 15 analytical datasets. The model with the lowest AIC is typically considered as the best. Table 2 reports the difference in AIC (ΔAIC) relative to the best-fitting model within each dataset. Following conventional guidance, a ΔAIC below about 2 indicates a model essentially equivalent to the best, values of roughly 4–7 indicate considerably less support, and values exceeding 10 indicate essentially no support of this model being equivalent to the best one^23^. The 4-par model consistently achieved the best fit (ΔAIC = 0) when the number of clinical malaria episodes exceeded 1000, and in these datasets its advantage over the best linear-in-parameter form was usually substantial: ΔAIC were frequently >4 and always >10 for Overall and Kenya pooled data. For datasets with fewer than 1000 episodes, the candidate models were typically separated by only a few ΔAIC units, indicating that the available data could not clearly distinguish among the functional forms.

**Table 2:**
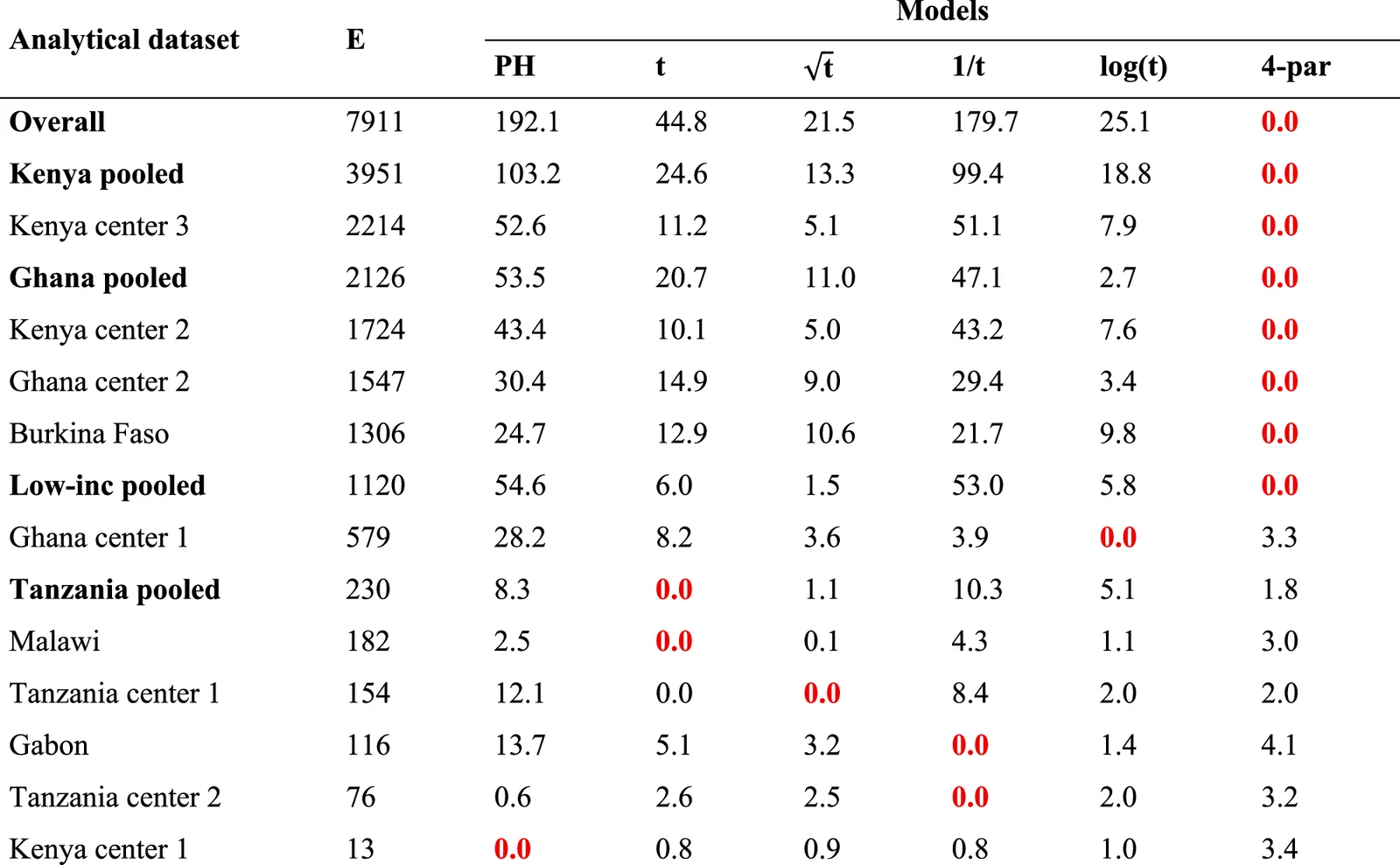
ΔAIC = AIC − lowest AIC within each dataset (10 individual sites, 4 pooled, and overall trial population)

Analytical datasets are ordered by descending *E* (number of clinical malaria episodes), with the pooled population shown in bold. The model with the lowest AIC is highlighted in red.

Detailed parameter estimates of the 4-par model for each analytical dataset are provided in Supplementary Table S1. Four parameters (details shown in “Methods”) are interpretable. The parameter *a* represents the effect immediately after vaccination (a more negative value corresponds to stronger initial protection); *b* controls the decline over time (a larger value corresponds to faster waning); *c* is the initial stability of the VE (a larger value corresponds to a longer initial plateau before VE begins to decline); and *d* represents the long-term effect (a more negative value corresponds to higher sustained long-term efficacy, positive values indicate potential harmful long-term effects)^15^. The parameter for the long-term effect *d* was negative in every analytical dataset except Ghana center 1. This center only has 579 episodes, which is well below the threshold at which the four-parameter model was favored, and confidence intervals of the 4-par model parameters were correspondingly large (Table S1), so the positive *d* at this single low-event site shall be interpreted as statistical variability under limited data instead of evidence of a genuine long-term harmful effect.

**Fig. 2:**
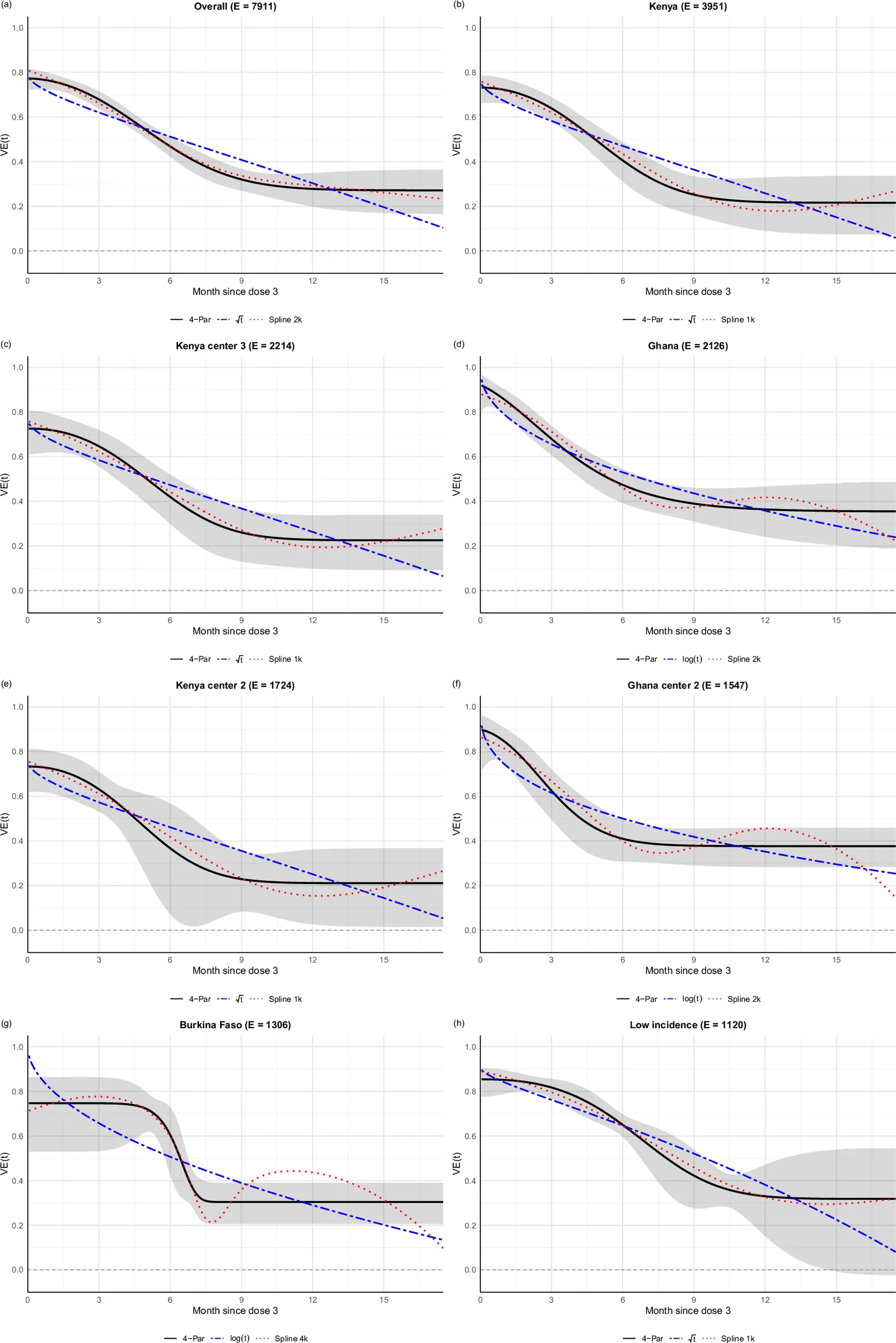
Time-varying RTS,S/AS01 vaccine efficacy in analytical datasets with a large number of clinical malaria episodes. **a**–**h** Estimated vaccine efficacy as a function of time for analytical datasets with more than 1000 clinical malaria episodes during the 18-month pre-booster period. Each panel corresponds to one analytical dataset: **a** overall trial population, **b** Kenya pooled, **c** Kenya center 3, **d** Ghana pooled, **e** Kenya center 2, **f** Ghana center 2, **g** Burkina Faso, and **h** pooled lower-incidence centers. The number of clinical malaria episodes in each dataset is shown in the panel title, denoted *E*. Within each panel, three curves are displayed: the simple linear-in-parameter or proportional hazards model with the lowest AIC (blue line), the four-parameter monotonic model (black line), and the natural cubic spline model with the lowest AIC (red dotted line, *k*: knots). The best-fitting parametric model (black line) is shown as a solid line, and its pointwise 95% confidence intervals are shown in shaded bands. The horizontal axis represents months since dose 3, and the vertical axis represents estimated time-varying VE. Across datasets with more than 1000 clinical malaria episodes, the four-parameter model closely follows the spline-based estimates while providing a smoother trajectory than simpler linear-in-parameter specifications.

**Fig. 3:**
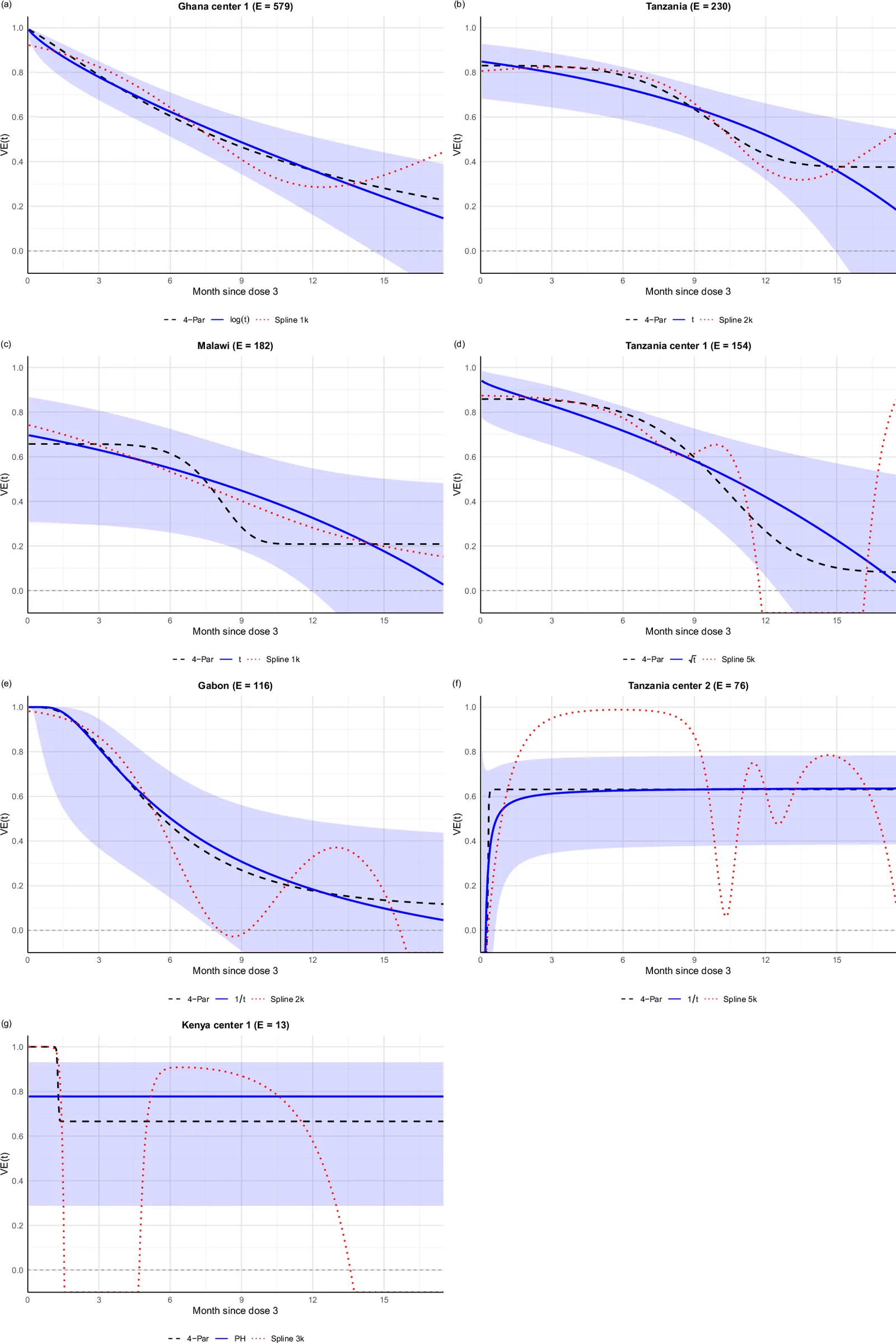
Time-varying RTS,S/AS01 vaccine efficacy in analytical datasets with a small number of clinical malaria episodes. **a**–**g** Estimated vaccine efficacy as a function of time for analytical datasets with less than 1000 clinical malaria episodes during the 18-month pre-booster period. Each panel corresponds to one analytical dataset: **a** Ghana center 1, **b** Tanzania pooled, **c** Malawi, **d** Tanzania center 2, **e** Gabon, **f** Tanzania center 1, and **g** Kenya center 1. The number of clinical malaria episodes in each dataset is shown in the panel title, denoted *E*. Within each panel, three curves are displayed: the simple linear-in-parameter or proportional hazards model with the lowest AIC (blue line), the four-parameter monotonic model (black line), and the natural cubic spline model with the lowest AIC (red dotted line, *k*: knots). The best-fitting parametric model (blue line) is highlighted as a solid line, and its pointwise 95% confidence intervals are shown in shaded bands. The horizontal axis represents months since dose 3, and the vertical axis represents estimated time-varying VE. In settings with fewer clinical malaria episodes, spline estimates exhibit greater variability, particularly toward the end of follow-up. Simpler linear-in-parameter or proportional hazards models are frequently favored by AIC, whereas the four-parameter model still provides trajectories that approximate the selected simpler functional forms.

To further examine model performance, we plotted the estimated VE(*t*) curves for settings with large (>1000, Fig. 2) and small (<1000, Fig. 3) numbers of clinical malaria episodes. For each analytical dataset, VE(*t*) curves estimated by three candidate specifications are shown: (i) the linear-in-parameter function or PH model with the lowest AIC (chosen from *t*, $\sqrt{{\rm{t}}}$, 1/*t*, log(*t*) or PH), (ii) the 4-par function, and (iii) the spline with number of knots that gave the lowest AIC (details of spline models in Table S2), used as a semi-parametric benchmark of the underlying efficacy trajectory. The best-performing parametric curve among (i) and (ii) is highlighted as a solid line for each analytical dataset, with their 95% confidence intervals shown in shaded bands. Observed monthly efficacy (VE = 1 − IRR), where the incidence rate ratio (IRR) compares clinical malaria episodes per person-time between the vaccine and control arms, is shown alongside the fitted curves in Supplementary Figs. S4 and S5.

For settings with a large number of clinical malaria episodes (>1000), the 4-par model not only achieved the best AIC but also captured VE(*t*) trajectories that closely approximated the spline curves (Fig. 2) and the observed monthly efficacy (Fig. S4), while smoothing out irregular fluctuations toward the end of follow-up. As the number of events increases, the 4-par model tends to align with spline trajectories and monthly efficacy more closely. This indicates that when sufficient event times data are available, the 4-par model can recover the underlying pre-booster VE(*t*) trajectory of RTS,S/AS01, with accuracy comparable to the flexible spline benchmark and greater parsimony and interpretability. The semi-parametric spline benchmark lay within the pointwise 95% confidence intervals (shading bands) across nearly all time points and analytical datasets, providing direct visual evidence that the 4-par model recovers the underlying VE(*t*) trajectory within sampling uncertainty.

For settings with a small number of clinical malaria episodes (<1000), linear-in-parameter functions were often favored by AIC (Table 2 and Fig. 3). As the number of events decreased, spline curves became unstable. Interestingly, even when the 4-par model was not favored by AIC, it could still capture trajectories that were close to the simple functional form selected. For example, in Ghana center 1 (Fig. 3a), the 4-par curve closely approximated the best-fitting linear-in-parameter curve, which was the log(*t*) curve. In the Gabon center, the 4-par curve closely approximated the best-fitting 1/*t* curve (Fig. 3e). Note that 1/*t* allows an initial stable efficacy period while log(*t*) does not, while the 4-par curve is flexible enough to capture both shapes.

### Vaccine impact in Burkina Faso

Among the RTS,S/AS01 vaccine study centers, Burkina Faso had the strongest seasonality of malaria transmission. We therefore used data from this center to evaluate how different specifications of modeling time-varying VE affect estimates of maximum vaccine impact, quantified by the number of cases averted under a seasonal vaccination strategy (${{NCA}}_{{season}}$), as defined in the “Methods.” Briefly, ${{NCA}}_{{season}}$ is derived from the estimated VE(*t*) curve through the area under the VE curve over the relevant follow-up period (AUC) and summarizes the total number of clinical malaria episodes prevented when the primary vaccination series is completed just prior to the high-transmission season.

Malaria disease incidence in Burkina Faso exhibits a sharp seasonal upsurge in June and subsides after December^24^. We found that if the target time of completing vaccination is just before June, ${{NCA}}_{{season}}$ per 1000 child-year varied substantially by different VE(*t*) specifications: 1786 for *t*, 1890 for $\sqrt{{\rm{t}}}$, 1456 for 1/*t*, 1944 for log(*t*), 2197 for the 4-par model, which fitted the data better than the linear-in-parameter functions, and 2225 for the spline benchmark (Tables 3 and S3). The 4-par model closely agreed with the spline benchmark. Using the linear-in-parameter model underestimated ${{NCA}}_{{season}}$ per 1000 child-year by 13% (log(*t*)) to 35% (1/*t*). As shown in Fig. 2g, the log(*t*) curve lies below the 4-par and spline curve during months 3–5 following vaccination, a period that coincides with peak clinical malaria incidence (Table 3, *k* = 3, 4, and 5). These months contributed disproportionately to the total number of cases averted, explaining the lower estimate of the log(*t*) specification.

**Table 3:** Estimated number of cases averted per 1000 child-year under a seasonal vaccination strategy for the Burkina Faso site (by months and in total for 1 year)

*k*	Month	*E*	*T*	1000 × *E*/*T*	AUC_*k* log_(*t*)	NCA_season_ log(*t*)	AUC_*k*_ 4-par	NCA_season_ 4-par	AUC_*k*_ spline	NCA_season_ spline
1	June	19	324.7	58.5	0.874	51.1	0.747	43.7	0.729	42.7
2	July	75	266.6	281.3	0.763	214.6	0.747	210.1	0.758	213.3
3	Aug	112	198.6	563.9	0.689	388.5	0.747	421.1	0.774	436.6
4	Sep	95	162.0	586.3	0.629	368.5	0.746	437.2	0.772	452.6
5	Oct	108	153.3	704.3	0.576	405.8	0.736	518.3	0.743	523.0
6	Nov	77	150.3	512.2	0.529	270.9	0.677	346.6	0.665	340.5
7	Dec	57	176.9	322.3	0.486	156.5	0.480	154.7	0.484	155.9
8	Jan	23	218.2	105.4	0.445	46.9	0.316	33.3	0.249	26.3
9	Feb	18	253.5	71.0	0.408	28.9	0.304	21.6	0.291	20.7
10	Mar	8	347.2	23.0	0.372	8.6	0.304	7.0	0.398	9.2
11	April	1	371.9	2.7	0.338	0.9	0.304	0.8	0.437	1.2
12	May	3	379.2	7.9	0.306	2.4	0.304	2.4	0.441	3.5
				**Total** **NCA**_**season**_		1944		2197		2225

VE(*t*) trajectory was specified using the best-fitting linear-in-parameter function (log(*t*)), the four-parameter monotonic function, and a spline with 4 interior knots. *k*: time in months since dose 3; *E*: number of clinical malaria episodes in the control group for each calendar month; *T*: person-time in the control group for each calendar month.

### Time-varying efficacy across settings

**Fig. 4:**
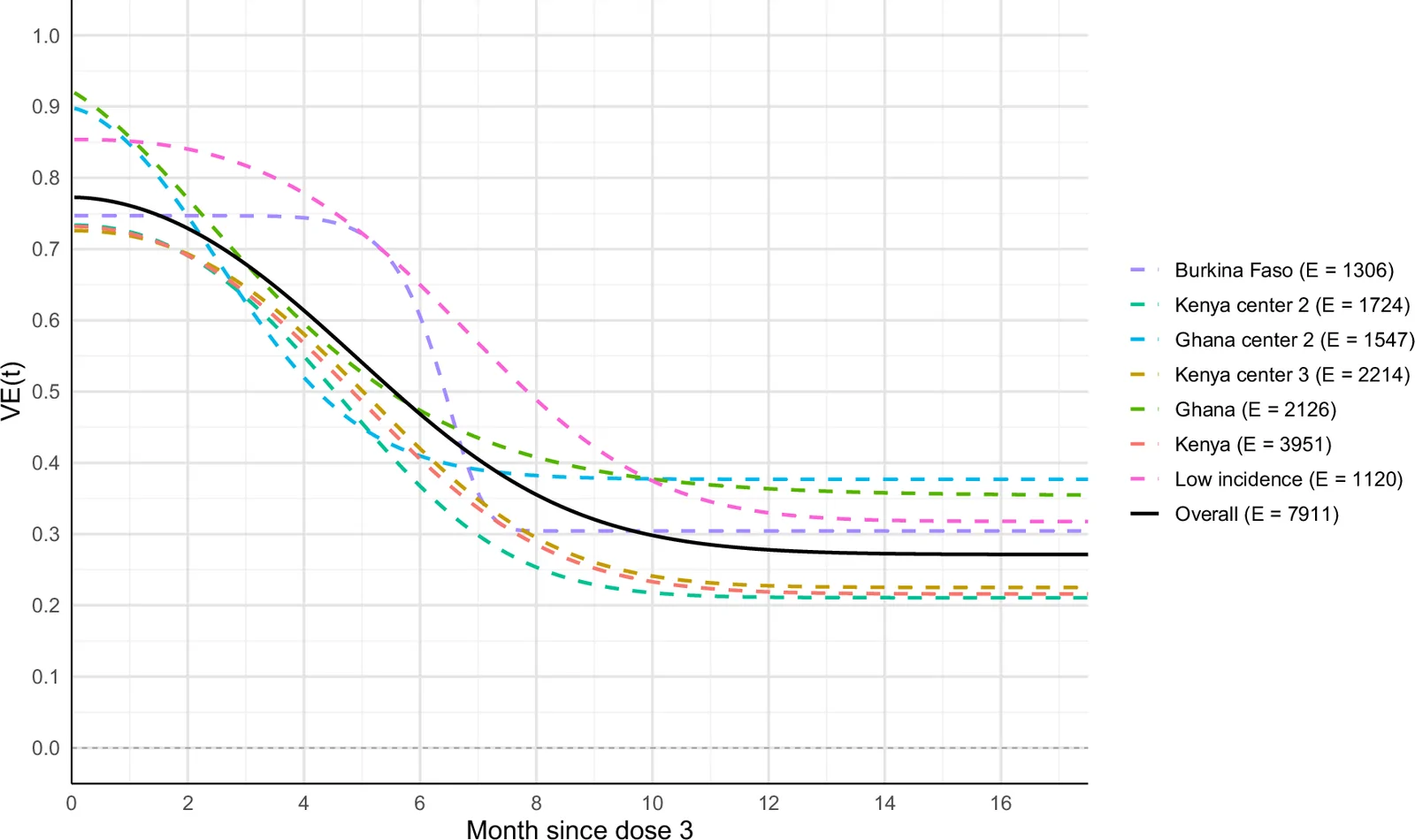
Four-parameter time-varying vaccine efficacy across settings with more than 1000 clinical malaria episodes. The figure shows estimated vaccine efficacy over time since completion of the primary three-dose series for all analytical datasets with more than 1000 clinical malaria episodes during the 18-month pre-booster period. The horizontal axis represents months since dose 3, and the vertical axis represents vaccine efficacy. Each colored line corresponds to the waning trajectory of efficacy estimated by the four-parameter monotonic model fitted to one analytical dataset, including four individual study centers (dashed), two pooled country datasets (dashed), the pooled low-incidence dataset (dashed), and the overall trial population (solid).

By plotting 4-par VE(*t*) curves for all settings with a large number of malaria episodes, we found similar patterns across centers, countries and the trial overall (Fig. 4). Despite geographic and epidemiological heterogeneity, these curves clearly followed a common inverse-sigmoid trajectory: VE was initially high (70–95%) shortly after completion of the primary three-dose series, declined during the first 6–8 months of follow-up, and then reached a relatively stable plateau of 20-40% by around 10 months. Previous statistical analysis estimating the RTS,S/AS01 vaccine efficacy used linear-in-parameter functional forms that go steadily downward and did not reveal this plateau. The pooled low-incidence analytical dataset showed a higher VE(*t*) over time compared with the overall population and the majority of other analytical datasets with higher disease incidence, which is consistent with observations in previous studies^1,25^.

## Discussion

Understanding how protection from malaria vaccination evolves after completion of the primary series remains a central challenge for interpreting vaccine durability and quantifying public health impact. For the RTS,S/AS01 malaria vaccine, efficacy is known to wane over time, but the temporal pattern of this waning was difficult to characterize consistently across trial settings. Commonly used simple model specifications impose restrictive assumptions on the pattern of waning efficacy, which may influence the interpretation of the long-term protection and the expected number of cases averted under a seasonal vaccination strategy. In this study, we systematically evaluated and compared the performance of four commonly used linear-in-parameter functions and a four-parameter monotonic function for modeling time-varying VE during the 18-month post-dose 3, pre-booster period of RTS,S/AS01. Across analytical datasets with a large number of clinical malaria episodes, the four-parameter model provided the best fit and enabled features of the efficacy trajectory to be assessed consistently across settings.

The flexible four-parameter monotonic model revealed a common temporal pattern of RTS,S/AS01 protection in settings with a large number of clinical malaria episodes. In these settings, VE was initially high, declined rapidly during the first several months after vaccination, and then stabilized at a lower level. However, the four-parameter model only achieved better model fit than simpler specifications when the number of clinical malaria episodes exceeded 1000, but not when the number of episodes was smaller. Two non-mutually exclusive explanations may account for this finding. One possibility is that the underlying efficacy trajectory of RTS,S/AS01 vaccine follows patterns supported by the four-parameter model only in places where malaria disease incidence is high. Another explanation is that datasets with a larger number of events provide sufficient information to support estimation of this more flexible parametric form, so the four-parameter form may be favored whenever the number of events is large. Although the RTS,S/AS01 trial did not include a single low-incidence site with a large number of events, the pooled analysis of low-incidence centers partially addressed this limitation. Despite a relatively low incidence, this pooled dataset contributed more than 1100 clinical malaria episodes, and the four-parameter model again provided a better fit. This suggests that a large number of events can support estimation of a more flexible efficacy trajectory even in lower-incidence settings. Further studies will be needed to confirm the observed efficacy pattern and whether it indeed does not vary by level of disease incidence.

In analytical datasets with fewer clinical malaria episodes, simpler linear-in-parameter specifications often achieved better model fit, but efficacy trajectories estimated using the four-parameter model closely approximated those obtained from the simpler specifications. This indicates that although simpler models may be statistically favored when data are limited, the four-parameter model is sufficiently flexible to mimic their shapes and adapt to such settings. As a result, the four-parameter model potentially provides a widely applicable parametric approach that facilitates comparison of time-varying VE across heterogeneous settings.

While using AUC as a summary measure of time-varying efficacy has been shown to be relatively robust to misspecification of the efficacy trajectory, our findings demonstrate that estimates of vaccine impact under a seasonal vaccination strategy (${{NCA}}_{{season}}$) can vary substantially depending on how the efficacy trajectory is specified. In Burkina Faso, where a highly seasonal malaria transmission is observed, different efficacy specifications led to considerably different estimates of ${{NCA}}_{{season}}$. Accurate specification of the efficacy curve is therefore particularly important when evaluating vaccine impact under a seasonal delivery strategy, and the four-parameter model provides a strong candidate for this purpose.

Our findings confirm and extend earlier characterizations of RTS,S/AS01 efficacy. Descriptive analyses of this trial reported an initial high level of protection followed by waning and efficacy rebound in high-transmission settings over longer follow-up^21,22^, as well as an inverse-sigmoid shape of protection over time predicted by antibody kinetic modeling^19^. The four-parameter form recovers these patterns empirically by time-to-event analysis, and its parameters provide an interpretable summary of them: the immediate effect, the rate of decline, the duration of initial stability, and the long-term level correspond qualitatively to features of the antibody decay process described in the antibody kinetic study^19^. The higher VE(*t*) observed over time in our pooled low-incidence dataset also accords with reports that efficacy is better sustained where transmission is less intense^25^. These results demonstrate that the four-parameter model is a statistically principled way to recover and describe this pattern consistently across heterogeneous settings.

Several methodological considerations are important when interpreting these findings. The Andersen–Gill framework used in this study estimates a composite effect that reflects both vaccine-induced protection and the accumulation of naturally acquired immunity^6,26^. RTS,S/AS01 is a pre-erythrocytic vaccine that acts against *P. falciparum* sporozoites before blood-stage infection is established, whereas naturally acquired anti-disease immunity develops through repeated blood-stage exposure^20^. Two processes that act in opposite directions affect the interpretation of the VE(*t*) curve. First, because children in the control group experienced more clinical malaria episodes than vaccinated children, they may have acquired natural immunity more rapidly, which would make the estimated composite effect of the vaccine appear smaller by comparison and waning faster over time. Second, vaccinated children remain exposed to sporozoites that the vaccine intercepts before they cause disease, and this exposure may boost vaccine-induced antibody responses, which would tend to preserve the estimated composite efficacy. In the four-parameter model, the parameter for the immediate effect (*a*) primarily reflects intrinsic vaccine protection shortly after dosing, before either process has developed, whereas the parameter for the long-term effect (*d*) captures their net result: a positive value of *d* would indicate that the natural-immunity differential has overtaken residual vaccine protection, producing a rebound in which efficacy declines and may eventually fall below zero. This effect is expected to be more pronounced in high-transmission settings. However, no evidence of such a rebound was observed in our 18-month analyses across sites with a large number of events, and the long-term parameter *d* remained negative throughout, indicating persistence of a protective effect over this period. The higher VE(*t*) observed over time in the pooled low-incidence dataset is consistent with this framework, since a weaker natural-immunity differential in lower-transmission settings would preserve the composite efficacy for longer.

To our knowledge, the four-parameter monotonic model has not previously been applied in VE studies. Our results demonstrate that this approach is flexible, parsimonious, interpretable, and can fit RTS,S/AS01 efficacy data well across large-event trial settings. Importantly, it can recover features of the efficacy trajectory that simpler linear-in-parameter models cannot capture, while still closely approximating those simpler forms when supported by the data. In doing so, this study adds evidence regarding the temporal pattern of RTS,S/AS01 efficacy following the primary vaccination series.

This study has limitations. Our analysis was restricted to the 18-month period prior to booster vaccination, and longer-term protection following booster dosing was not examined. Modeling efficacy across multiple doses would require accommodating non-monotonic effects and warrants future investigation. Extending this framework to connect different phases of long-term VE before and after booster vaccination will be an important direction for future work. Another limitation is that the four-parameter function was evaluated here only for RTS,S/AS01. The broader methodological approach that combines an interpretable parametric form with AUC summaries and information-criterion-based model comparison is in principle applicable to other vaccines, but whether the four-parameter function is also favored for vaccines with different mechanisms or waning dynamics will require evaluation in other settings.

## Methods

### RTS,S/AS01 malaria vaccine trial

We conducted a re-analysis of data from the phase 3 randomized controlled trial of the RTS,S/AS01 malaria vaccine. The trial was conducted across 11 African centers in seven countries, and subjects were recruited between March 2009 and January 2011. Data from 10 centers were available for per-protocol analysis. Children aged 5 to 17 months and infants aged 6–12 weeks were randomized in a 1:1:1 ratio to one of three arms: three doses of RTS,S/AS01 at months 0, 1, and 2 followed by a booster at month 20 (R3R); three doses at months 0, 1, and 2 followed by a comparator at month 20 (R3C); or four doses of comparator at months 0, 1, 2, and 20 (C3C)^1^.

The RTS,S Clinical Trials Partnership reported that young infants responded less effectively to RTS,S/AS01 than children^1,27^, so our analysis focused on the children cohort. To evaluate the efficacy of the three-dose primary series, we combined participants in the R3R and R3C groups to form the intervention arm and compared them with the C3C control arm. The per-protocol population included 4532 children in the intervention arm and 2319 in the control arm. Follow-up for efficacy assessment began 14 days after the third dose and continued for 18 months, ending just prior to administration of the scheduled booster^1^. We restricted the analysis to this pre-booster period to focus on waning protection following the primary three-dose series; modeling post-booster efficacy would require accommodating non-monotonic effects and is beyond the scope of this study. The outcome event was clinical malaria caused by *P. falciparum*, defined as in the original RTS,S/AS01 trial: an illness with an axillary temperature ≥37.5 °C and *P. falciparum* asexual parasitaemia >5000 parasites/μL, or a case meeting the primary case definition of severe malaria^1^.

### Modeling time-varying VE

The standard Cox proportional hazard model^4^ assumes a constant intervention effect:

$${\lambda }_{i}\left(t\right)={\lambda }_{0}\left(t\right)\exp \left(\beta {z}_{i}\right)$$

where ${\lambda }_{0}\left(t\right)$ is the unspecified baseline hazard, ${z}_{i}$ is the intervention status of subject *i* (1 = vaccine, 0 = control), and *β* is the log(HR) to be estimated. The VE is then estimated as $\hat{{\rm{VE}}}=1-\exp \left(\hat{\beta }\right)$ and is constant over time.

To allow waning intervention effect, the hazard function for the *i*th person can be extended by introducing a time-varying effect function $g\left(t\right)$:

$${\lambda }_{i}\left(t\right)={\lambda }_{0}\left(t\right)\exp \left[g\left(t\right){z}_{i}\right].$$

The time-varying HR of the intervention arm over the control arm is given by ${\rm{HR}}\left(t\right)=\frac{\lambda (t| {z}_{i}=1)}{\lambda (t| {z}_{i}=0)}=\exp \left[g\left(t\right)\right]$, and the corresponding ${\rm{VE}}\left(t\right)$ is:

1 $${\rm{VE}}\left(t\right)=1-{\rm{HR}}\left({\rm{t}}\right)=1-\exp \left[g\left(t\right)\right]$$

where specifications of *g*(*t*) correspond to different assumptions about how protection wanes over time and determine the shape of the VE(*t*) trajectory.

Since infectious diseases like clinical malaria can occur recurrently, we used the Andersen–Gill extension of the Cox model^5^. The value of the AG model in the estimation of VE had been discussed previously^6,26^.

For each of the analytical datasets defined in Table 1, besides the model assuming PH, we evaluated several candidate parametric specifications of *g*(*t*) that assume a monotonic decline in VE(*t*) after completion of the primary series, including 4 simple linear-in-parameter forms that have been used by the RTS,S study team (functions 1 to 4) and the four-parameter monotonic function (4-par) (function 5):

1. $${{g}_{t}\left(t\right)=\beta }_{0}+{\beta }_{1}t$$
2. $${{g}_{\sqrt{t}}\left(t\right)=\beta }_{0}+{\beta }_{1}\sqrt{{\rm{t}}}$$
3. $${{g}_{1/t}\left(t\right)=\beta }_{0}+{\beta }_{1}\frac{1}{t}$$
4. $${{g}_{\log (t)}\left(t\right)=\beta }_{0}+{\beta }_{1}\log t$$
5. $${g}_{4-{\rm{par}}}(t)=a\exp \left(-b{t}^{c}\right)+d$$

In ${g}_{4-{\rm{par}}}(t)$, the four parameters are interpretable. The parameter *a* represents the effect immediately after vaccination, *b* controls the decline over time of VE, *c* is the initial stability of the VE, and *d* represents the long-term effect, with positive values indicating potential harmful long-term effects^15^.

Natural cubic splines with one to five interior knots were also fitted as flexible, semi-parametric benchmarks for the underlying VE(*t*) trajectory. These fits are intended as references for assessing whether the parametric forms sufficiently capture features of the data. Interior knots were placed at quantiles of observed event times (e.g., one knot at the median of event times; two knots at the 33rd and 67th percentiles; and so on)^28^.

Each of the estimated *g*(*t*) can be substituted into Eq. (1) and give a VE(*t*) curve. For example, if we take ${g}_{4-{par}}\left(t\right)=a\exp \left(-b{t}^{c}\right)+d$, then Eq. (1) can be expanded to ${\rm{VE}}\left(t\right)=1-{\rm{HR}}\left({\rm{t}}\right)=1-\exp [a\exp \left(-b{t}^{c}\right)+d]$. The model for pooled analysis of multiple centers of a country and overall analyses pooling all centers were stratified by study center. Stratification allows a separate baseline hazard per stratum. We used AIC as a criterion to compare the model fits.

### AUC and number of cases averted

To evaluate how different time-varying efficacy trajectories affect summary efficacy and policy-relevant measures of vaccine impact, we estimated both the area under the VE curve (AUC) and the corresponding number of cases averted (NCA), with NCA derived from the estimated VE(*t*) curve via AUC.

${{AUC}}_{{t}_{1}-{t}_{2}}$ quantifies the area under VE(*t*) during an interval of time from *t*_1_ to *t*_2_:

$${{AUC}}_{{t}_{1}-{t}_{2}}=\frac{{\int }_{{t}_{1}}^{{{t}_{2}}}{\rm{VE}}\left(t\right){dt}}{{t}_{2}-{t}_{1}}$$

In previous studies, several approaches have been used to estimate NCA. The conventional method is based on empirically estimated step functions of incidence rate over time^29,30^. A recently proposed approach estimates ${NC}{A}_{{AUC}}$ from incidence data of the control arm using interval-based AUC, which is more flexible and reduces sensitivity to background incidence and timing of vaccination. In this study, we focus on ${{NCA}}_{{season}}$ that extends the ${NC}{A}_{{AUC}}$ approach to evaluate the potential impact of aligning vaccination with seasonal peaks in transmission^7^. This approach assumes that completion of the vaccination for all subjects is right before the start of the high-transmission season, and the NCA per 1000 child-year can be estimated as:

$${{NCA}}_{{season}}=1000\mathop{\sum }\limits_{k=1}^{12}{{AUC}}_{k}\left(\frac{{E}_{s+k-1}}{{T}_{s+k-1}}\times \Delta {tim}{\rm{e}}\right)$$

where ${{AUC}}_{k}$ is the area under VE(*t*) curve during the *k*th month since completion of vaccination, $\varDelta {tim}{\rm{e}}$ is the duration of the interval and is one month here, *s* denotes the target month before which vaccination campaign is to complete, e.g., *s* = 6 if the target is to complete vaccination immediately before June, and ${E}_{s+k-1}$ and ${T}_{s+k-1}$ are the number of outcome events and person-time in the control group for each interval respectively. In this analysis, ${E}_{s+k-1}$ and ${T}_{s+k-1}$ were computed from the pooled calendar-month incidence data across all observed years of the trial.

All analyses were performed in R version 4.4.0. PH and linear-in-parameter time-varying models were fitted using the coxph function in the survival package, with time-varying coefficients specified through the tt argument; natural cubic splines were constructed using the ns function in the splines package, with interior knots placed at quantiles of the observed event times. The four-parameter monotonic model was fitted by maximizing the corresponding likelihood using the optim function with the BFGS algorithm, retaining the Hessian for variance estimation. Robust sandwich standard errors were used throughout to account for within-subject correlation among recurrent episodes. AIC was computed as −2 × log-likelihood + 2 × *k*, where the log-likelihood is the maximized partial likelihood for the Andersen–Gill Cox-type models (PH, linear-in-parameter, and splines via AIC() on the coxph fits, with *k* equal to the number of regression coefficients) and the maximized full custom log-likelihood for the four-parameter model (with *k* = 4). Codes for the 4-par model are available at https://github.com/cheungyb/PE-monotonic. Codes for re-analyzing the RTS,S/AS01 data are available at https://github.com/ZiweiZhao99/RTS-S-AS01_2026.

To quantify uncertainty in the estimated VE(*t*) curves, we computed pointwise 95% confidence intervals using the delta method applied to the robust sandwich covariance matrix appropriate for the Andersen–Gill model with within-subject clustering^31^. For the four-parameter model, the variance of the estimated VE at each time point was obtained from the gradient of VE with respect to the parameters and the estimated parameter covariance matrix. Confidence limits were computed on the log-hazard-ratio scale and back-transformed to the VE scale. Pointwise variances for the linear-in-parameter and spline models were obtained analogously from the corresponding design vectors. Observed monthly incidence rate ratios were computed as the ratio of clinical malaria incidence (events per person-time) in the vaccine arm to the control arm in each post-dose-3 month, with 95% confidence intervals from the standard log-IRR normal approximation. The cumulative number of clinical malaria episodes per child in each arm was estimated using the Nelson-Aalen estimator for recurrent events, with pointwise 95% confidence intervals.

### Ethics declaration

The original RTS,S/AS01 phase 3 trial was conducted in accordance with the Declaration of Helsinki and Good Clinical Practice guidelines, and was approved by the institutional review boards or national ethics committees at each of the 11 participating sites in the seven African countries, with full details and approval numbers reported in the primary trial publications^1,27^. Written informed consent was obtained from each child’s parent or legal guardian during the original trial. The present study is a secondary analysis of de-identified trial data accessed through Vivli (#GSK-110021) and did not involve new participant recruitment; the secondary analysis was approved by the National University of Singapore Institutional Review Board (NUS-IRB-2022-510), and the need for additional informed consent was waived because only de-identified data were analyzed.

## Supplementary information

Supplementary Materials

## Data Availability

The RTS,S/AS01 trial data is owned by GlaxoSmithKline. The authors obtained access to the data (#GSK-110021) via application to and permission by www.clinicalstudydatarequest.com. A request for access to the data may be submitted to www.clinicalstudydatarequest.com for the data owner’s consideration.

## Code availability

R codes for the model estimation and data analysis are available at https://github.com/cheungyb/PE-monotonic and https://github.com/ZiweiZhao99/RTS-S-AS01_2026.
