# Supplementary material for: Modeling time-varying efficacy of RTS,S/AS01 malaria vaccine across trial settings
Source: NPJ Vaccines. 2026 Jul 3;11:196. doi: 10.1038/s41541-026-01519-4 (PMC13696702; doi:10.1038/s41541-026-01519-4)
Supplement: Supplementary file 1 [file 41541_2026_1519_MOESM1_ESM.pdf]

Supplemental Materials

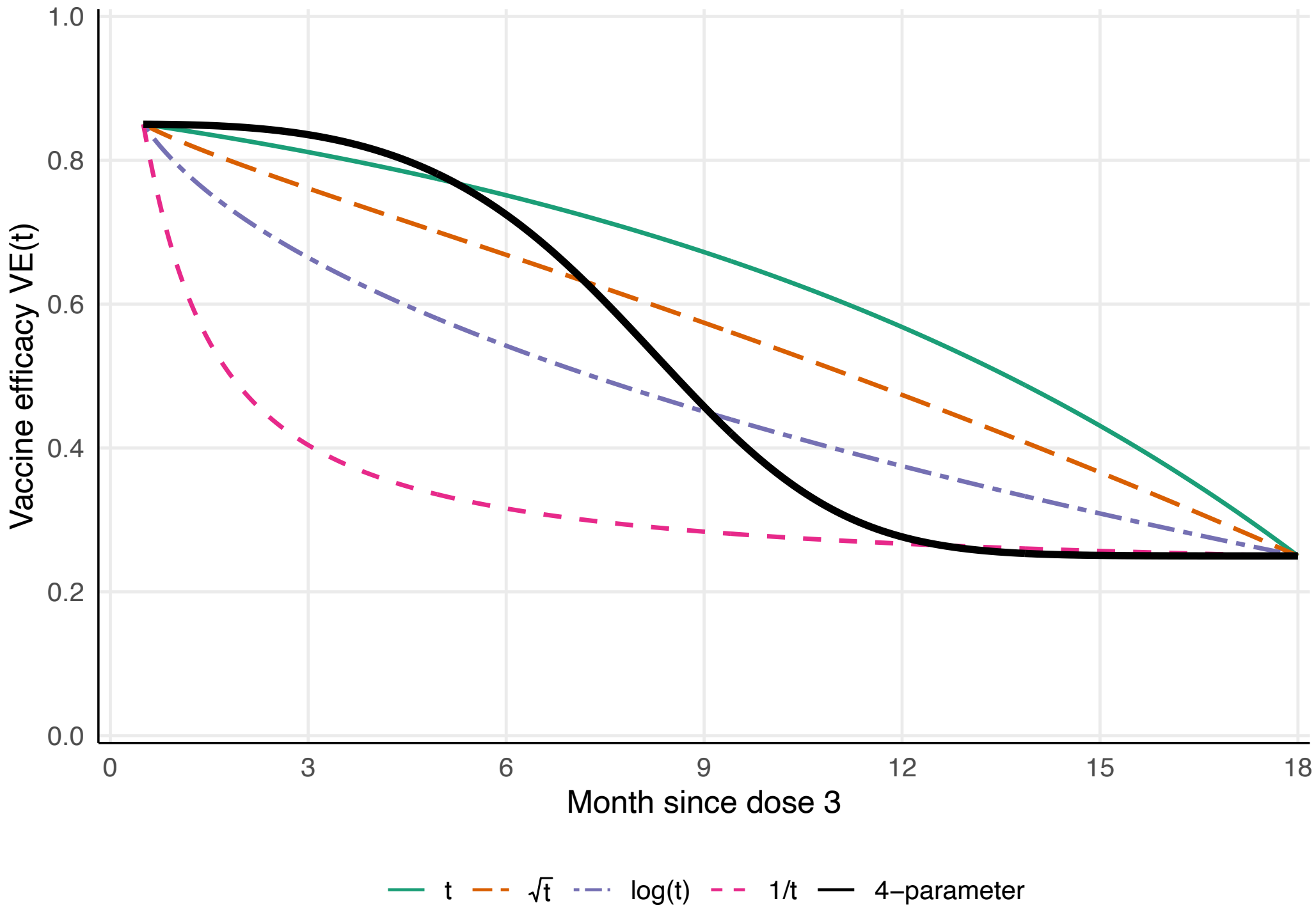

Figure S1 | Illustrative shapes of the candidate VE(t) functional forms. For the linear-in-parameter forms, $g(\mathrm{t}) = \beta_0 + \beta_1 f(\mathrm{t})$ with $f(t) = t$, $\sqrt{\mathrm{t}}$, $\log(t)$, or $1/\mathrm{t}$; coefficients were set so all four share an initial VE of 0.85 at month 0.5 and VE of 0.25 at month 18, isolating differences in shape (t: $\beta_0 = -1.94$, $\beta_1 = 0.092$; $\sqrt{\mathrm{t}}$: −2.22, 0.455; $\log(t)$: −1.59, 0.449; $1/\mathrm{t}$: −0.24, −0.828). The four-parameter function $g(\mathrm{t}) = a\exp(-bt^c) + d$ is shown with $a = -1.61$, $b = 0.0022$, $c = 3$, $d = -0.29$.

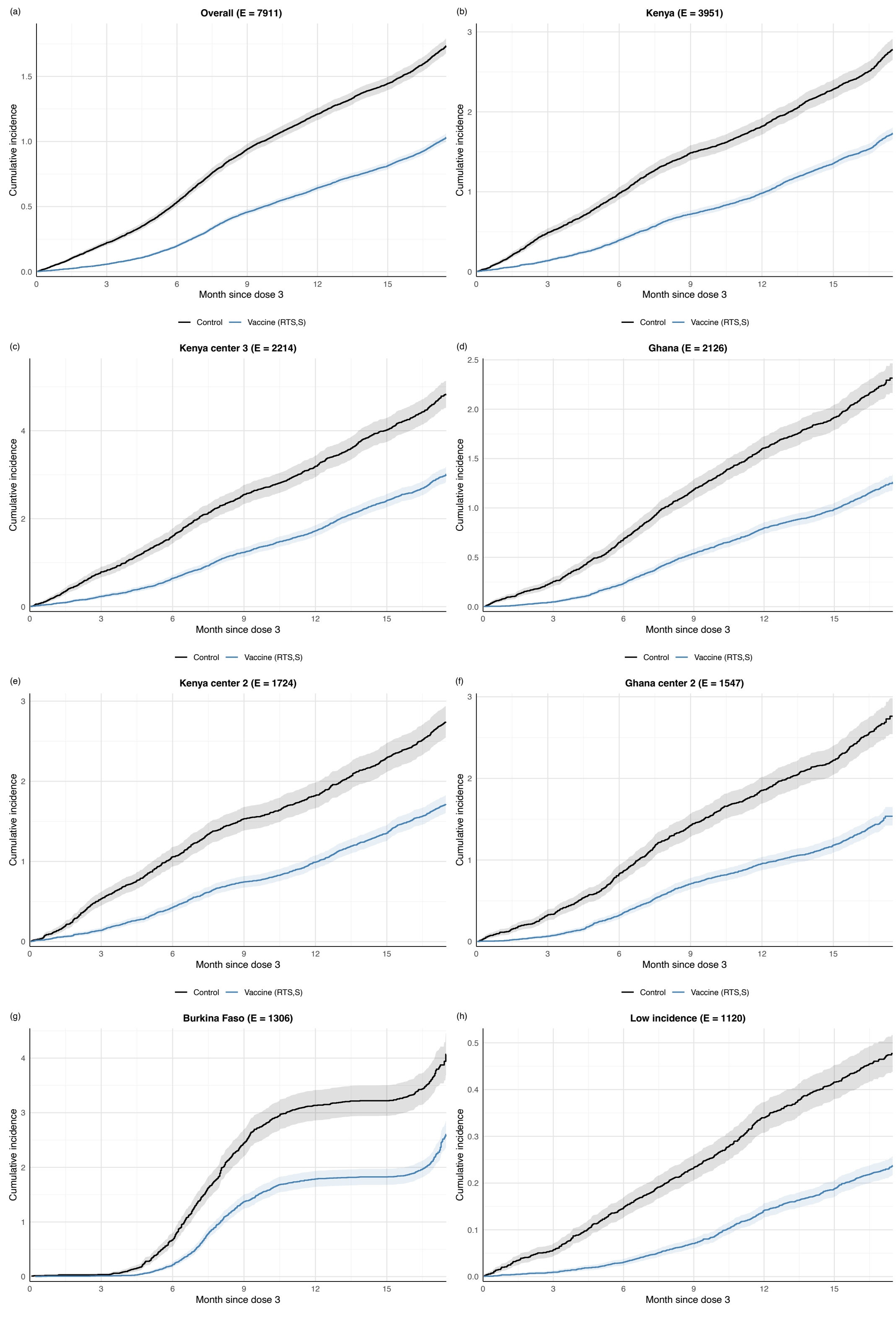

Figure S2 | Nelson-Aalen cumulative incidence plots showing the mean cumulative number of clinical malaria episodes per child by trial arm for large-event datasets.

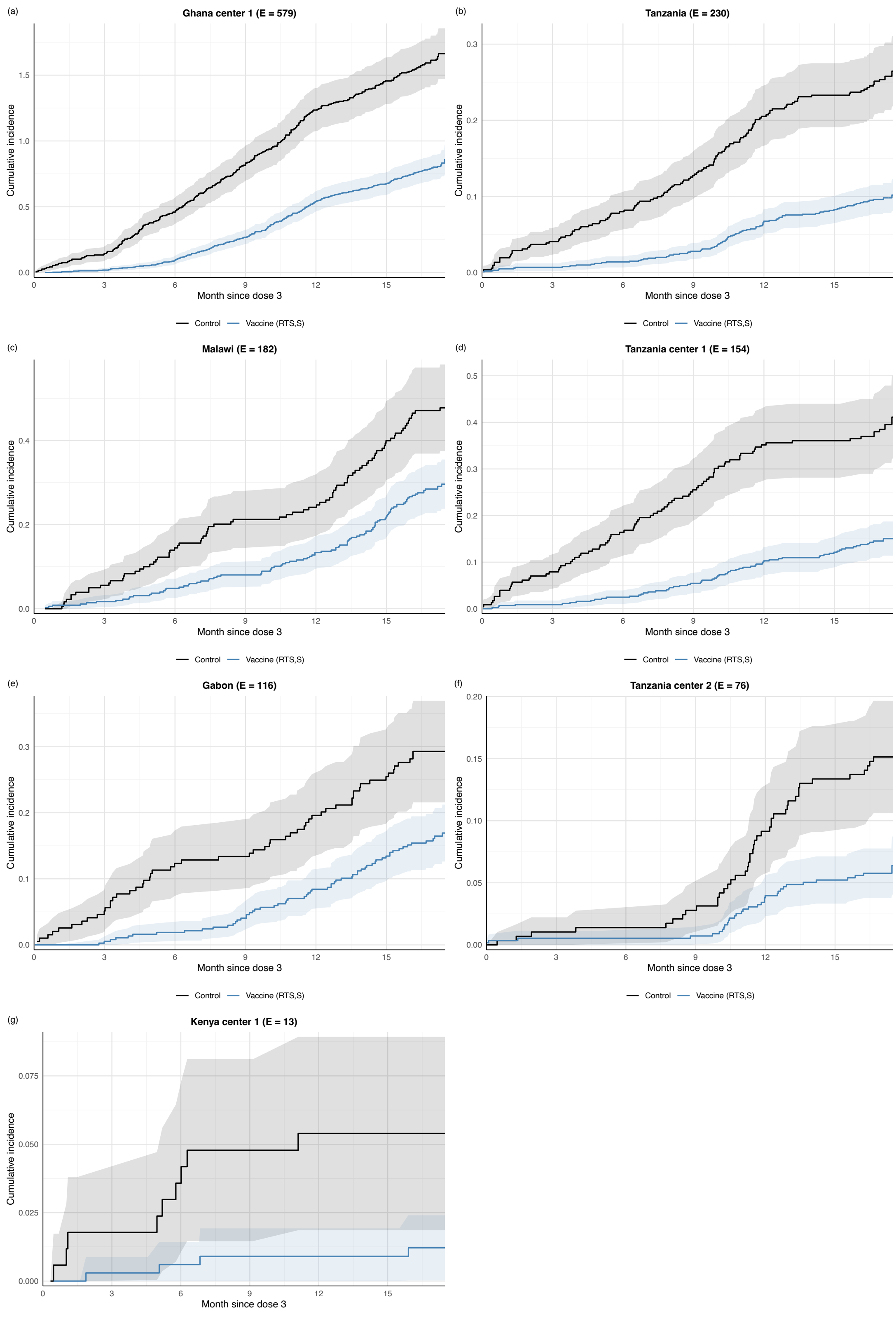

Figure S3 | Nelson-Aalen cumulative incidence plots showing the mean cumulative number of clinical malaria episodes per child by trial arm for small-event datasets.

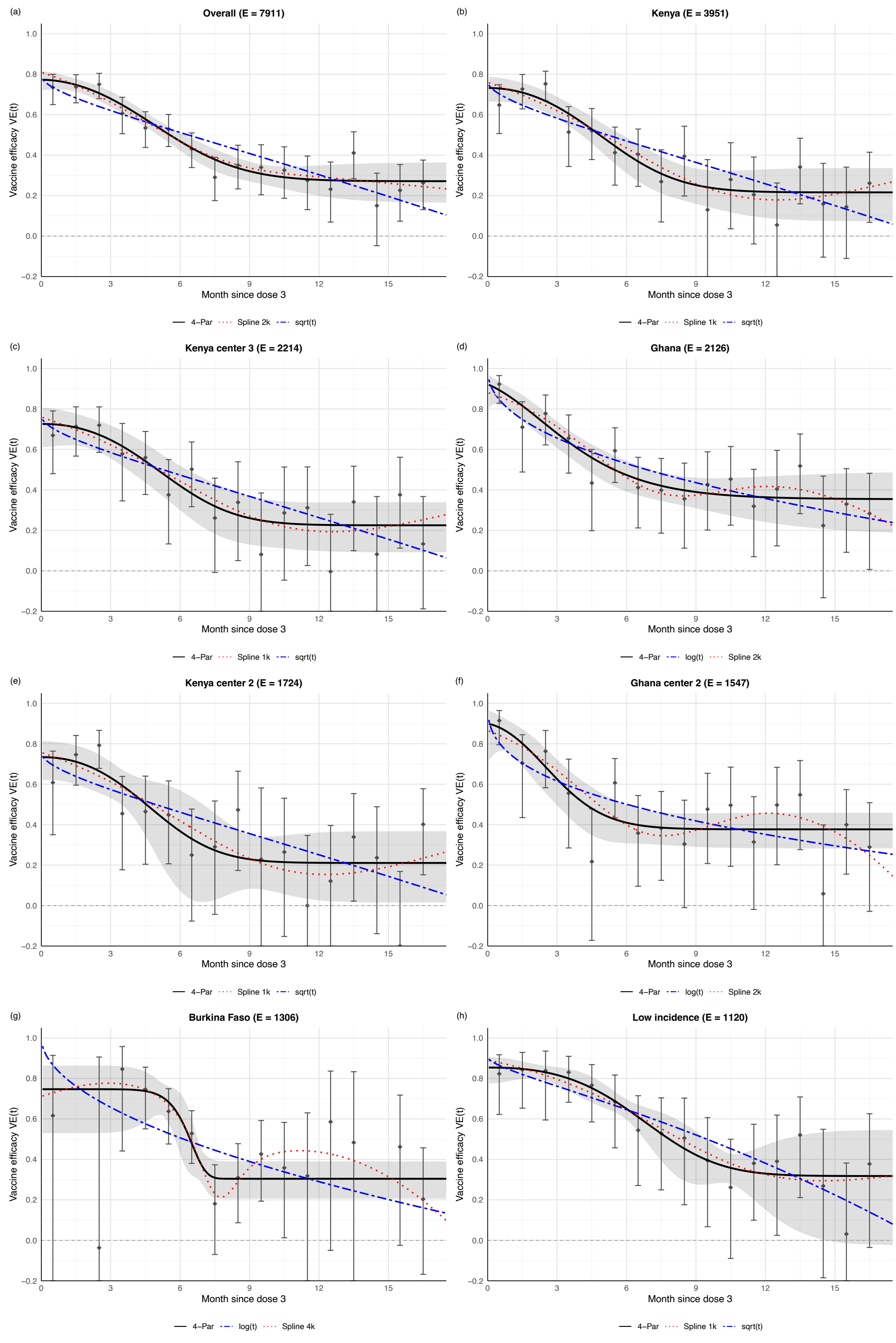

Figure S4 | Time-varying RTS,S/AS01 vaccine efficacy curves and observed monthly efficacy (with 95% error bars) in analytical datasets with large number of clinical malaria episodes. Some monthly efficacy bars are missing because event counts are too small.

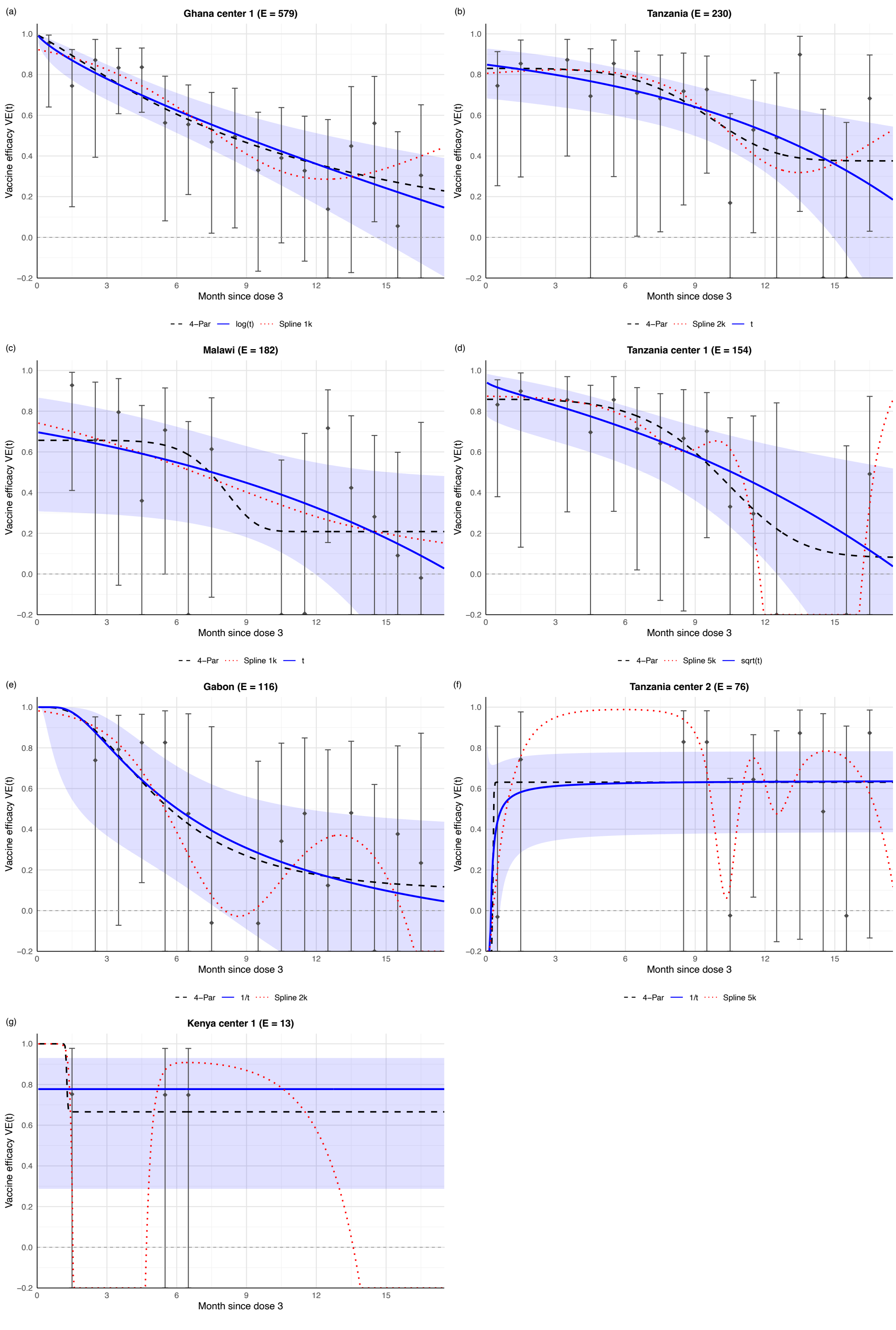

Figure S5 | Time-varying RTS,S/AS01 vaccine efficacy curves and observed monthly efficacy (with 95% error bars) in analytical datasets with small number of clinical malaria episodes. Some monthly efficacy bars are missing because event counts are too small.

Analytical dataset	E	a (SE)	b (SE)	c (SE)	d (SE)
**Overall**	7911	-1.165 (0.143)	0.044 (0.036)	1.891 (0.501)	-0.317 (0.070)
**Kenya pooled**	3951	-1.073 (0.143)	0.034 (0.024)	2.058 (0.454)	-0.243 (0.085)
Kenya center 3	2214	-1.039 (0.184)	0.025 (0.037)	2.187 (0.774)	-0.255 (0.081)
**Ghana pooled**	2126	-2.109 (0.559)	0.335 (0.221)	1.082 (0.437)	-0.437 (0.122)
Kenya center 2	1724	-1.086 (0.192)	0.033 (0.047)	2.168 (1.099)	-0.237 (0.113)
Ghana center 2	1547	-1.810 (0.537)	0.254 (0.248)	1.465 (0.664)	-0.473 (0.072)
Burkina Faso	1306	-1.011 (0.311)	1.9e-08 (2.1e-07)	9.607 (5.441)	-0.363 (0.067)
**Low-inc pooled**	1120	-1.540 (0.387)	0.011 (0.030)	2.413 (1.537)	-0.382 (0.207)
Ghana center 1	579	-6.265 (5.232)	0.833 (0.673)	0.441 (0.245)	0.072 (0.582)
**Tanzania pooled**	230	-1.302 (0.785)	2.3e-04 (0.001)	3.749 (3.344)	-0.471 (0.709)
Malawi	182	-0.836 (0.982)	1.3e-06 (1.2e-04)	6.491 (37.744)	-0.234 (0.268)
Tanzania center 1	154	-1.869 (0.616)	5.9e-04 (0.004)	3.295 (2.662)	-0.085 (0.387)
Gabon	116	-13.734 (12.206)	1.081 (1.008)	0.613 (0.406)	-0.098 (0.369)
Tanzania center 2	76	8.178 (0.761)	246.719 (81.119)	3.908 (0.387)	-0.997 (0.263)
Kenya center 1	13	-9.173 (0.893)	0.054 (0.011)	16.005 (1.719)	-1.095 (0.637)

Table S1 | Coefficients for four-parameter monotonic function $g(t) = a\exp(-bt^c) + d$ across all analytical datasets. a: immediate effect; b: rate of decline; c: initial stability; d: long-term effect (a positive value would indicate a harmful long-term effect). Standard errors are on the natural parameter scale.

Analytical dataset	Spline Settings				
	**1 knot**	**2 knots**	**3 knots**	**4 knots**	**5 knots**
**Overall**	101636.4	**101635.1**	101637.2	101636.9	101638.9
**Kenya pooled**	**51324.3**	51326.1	51328.2	51329.6	51330.6
Kenya center 3	**28205.5**	28207.5	28209.2	28210.7	28212.6
**Ghana pooled**	27748.9	**27742.7**	27743.9	27745.8	27747.7
Kenya center 2	**22968.6**	22970.5	22972.1	22974.1	22974.7
Ghana center 2	20596.4	**20588.5**	20589.6	20623.4	20592.5
Burkina Faso	15901.6	15901.3	15895.7	**15893.5**	15894.0
Low-inc pooled	**13954.9**	13957.0	13958.6	13959.6	13961.1
Ghana center 1	**7149.5**	7151.2	7153.2	7155.2	7157.0
**Tanzania pooled**	2953.3	**2952.8**	2954.7	2956.0	2956.5
Malawi	**2259.6**	2261.5	2261.8	2262.3	2265.3
Tanzania center 2	1944.6	1944.7	1944.6	1943.7	**1943.4**
Gabon	**1446.4**	1445.4	1447.7	1449.2	1450.3
Tanzania center 1	1010.4	1012.2	1012.6	1013.0	**1010.3**
Kenya center 1	159.1	159.6	**158.3**	160.0	161.3

Table S2 | AIC of models based on splines. For each analytical dataset, the lowest AIC is shown in bold and highlighted in red.

k	Month	1000*E/T	AUC_k t	NCA_{season} t	AUC_k $\sqrt{t}$	NCA_{season} $\sqrt{t}$	AUC_k 1/t	NCA_{season} 1/t	AUC_k log(t)	NCA_{season} log(t)
1	June	58.5	0.640	37.4	0.752	44.0	0.746	43.7	0.874	51.1
2	July	281.3	0.618	173.9	0.695	195.6	0.522	146.8	0.763	214.6
3	Aug	563.9	0.596	336.1	0.651	367.0	0.469	264.2	0.689	388.5
4	Sep	586.3	0.572	335.5	0.610	357.8	0.445	260.8	0.629	368.5
5	Oct	704.3	0.547	385.3	0.571	402.4	0.431	303.8	0.576	405.8
6	Nov	512.2	0.520	266.6	0.533	273.2	0.423	216.5	0.529	270.9
7	Dec	322.3	0.492	158.6	0.496	159.7	0.417	134.3	0.486	156.5
8	Jan	105.4	0.462	48.7	0.458	48.3	0.412	43.4	0.445	46.9
9	Feb	71.0	0.421	30.6	0.420	29.9	0.409	29.0	0.408	28.9
10	Mar	23.0	0.397	9.2	0.383	8.8	0.406	9.4	0.372	8.6
11	April	2.7	0.362	1.0	0.344	0.9	0.404	1.1	0.338	0.9
12	May	7.9	0.324	2.6	0.306	2.4	0.402	3.2	0.306	2.4
		**Total NCA_{season}**		1786		1890		1456		1944

Table S3 | Estimated number of cases averted under a seasonal vaccination strategy for the Burkina Faso site shown in monthly intervals. VE(t) trajectory was specified using simple linear-in-parameter forms.
